# Development and validation of a nomogram prediction model for oral frailty in community-dwelling older adults

**DOI:** 10.3389/fpubh.2026.1793603

**Published:** 2026-04-28

**Authors:** Wenjuan Yang, Qin Pan, Qingfang Lin, Liping Yang, Juan He, Junna Wang, Xia Zhang, Daiying Jing, Kangqin Cai, Meng Fang

**Affiliations:** Zigong First People's Hospital, Zigong, Sichuan, China

**Keywords:** age, oral frailty, risk indicators, nomogram, prediction model

## Abstract

**Objective:**

This study aims to investigate the factors influencing oral frailty among community-dwelling older adults and to develop and validate a risk prediction model.

**Methods:**

A cross-sectional study was conducted between October 2023 and November 2024, enrolling community-dwelling older adults from Zigong City, Sichuan Province, China. Participants were randomly allocated into a training set (*n* = 271) and a validation set (*n* = 117) in a 7:3 ratio. Variable selection was performed using Lasso regression, followed by multivariate logistic regression to develop a risk prediction model, which was presented as a nomogram. The model’s goodness-of-fit and predictive performance were assessed using the Hosmer–Lemeshow (H-L) test and the receiver operating characteristic (ROC) curve, respectively. Internal validation was further carried out via 1,000 bootstrap resamples. ROC curves, decision curve analysis (DCA), and calibration curves were plotted to comprehensively evaluate the predictive performance of the nomogram.

**Result:**

The prevalence of oral frailty among community-dwelling older adults was 32.5%. Logistic regression analysis identified several independent risk factors for OF (all *p <* 0.05), including sex, age, education level, number of chronic diseases, smoking status, number of natural teeth, difficulty chewing hard foods, frailty status, and oral health-related self-efficacy. The prediction model demonstrated excellent performance, with an area under the ROC curve (AUC) of 0.945 (95% *CI*: 0.919–0.970), a sensitivity of 0.828, and a specificity of 0.913 in the training set. Internal validation using the validation set yielded an AUC of 0.910 (95% *CI*: 0.857–0.962), a sensitivity of 0.923, and a specificity of 0.808, indicating robust model performance.

**Conclusion:**

The occurrence of oral frailty among community-dwelling older adults is influenced by multiple factors including sex, age, difficulty chewing hard foods, and oral health-related self-efficacy. The risk prediction model constructed based on these factors demonstrates favorable discrimination and calibration, with its predictive performance being adequately validated. This model can serve as an evidence-based practical tool to support early screening, prevention, and the formulation of individualized intervention strategies for oral frailty in community settings.

## 1 Introduction

As the global aging population continues to accelerate, promoting healthy aging among the older adults has become a core issue in public health. According to surveys, China’s population aged 60 and above will exceed 20% of the total population, marking the country’s entry into a moderately aged society. Within the realm of older adult health, the close connection between oral health and overall physical wellbeing, nutritional status, and quality of life among seniors is increasingly evident. Oral health, as a key factor in healthy aging, is often overlooked (1). The findings of China’s Fourth National Oral Health Epidemiological Survey reveal that among adults aged 65–74, only 9.3% maintain healthy gums, and just 18.3% retain a full set of teeth. Furthermore, the National Health Commission’s “Healthy Mouth Action Plan (2019–2025)” (2) designates the number of remaining teeth in individuals aged 65–74 as one of its key performance indicators for oral health initiatives. China’s first blue book on geriatric oral health, the China Oral Health Development Report (2022)-Oral Health Status of the Older Adults (3), published in 2022, highlights that oral health issues among the older adults are more complex. Not only do oral diseases have a high incidence rate among this demographic, but they also exhibit a stronger correlation with systemic diseases, exerting a greater impact on overall health. This directly affects the quality of life, life expectancy, and survival status of the older adults. Therefore, prioritizing the oral health of the older adults is a critical task at present.

Oral frailty (OF), as an emerging comprehensive concept, specifically refers to the persistent decline in oral function caused by multiple factors such as tooth loss, weakened chewing ability, reduced swallowing function, decreased saliva secretion, oral dryness, and oral microbial imbalance. This decline subsequently leads to a series of adverse health outcomes including nutritional intake disorders, sarcopenia, cognitive decline, and reduced social participation (4). Oral frailty not only signifies localized oral functional decline but also serves as a critical precursor indicator for predicting systemic frailty, disability, and mortality in older adults (5). Epidemiological studies indicate a prevalence of OF among community-dwelling older adults in Japan ranging from 8.4 to 49.4% (4, 6–9). Studies by Lin (10) and Tu et al. (11) found community-dwelling older adults OF prevalence rates of 20.7 and 33.8%, respectively. A 6-year longitudinal study demonstrated that older adults with OF had higher probabilities of disability or mortality, and incurred greater healthcare expenditures for adverse health outcomes attributable to OF compared to healthy older adults (12). Therefore, early screening and identification, coupled with targeted interventions, are essential to shift from reactive treatment to proactive prevention, thereby reducing or delaying the onset and progression of OF in community-dwelling older adults.

However, in community settings, older adults often dismiss the gradual decline in oral function as a “normal aging process” and neglect it. Traditional oral health examinations focus on diagnosing diseases like dental caries and periodontitis, lacking early, systematic screening for overall oral function status and its risk of deterioration. This results in many older adults only seeking intervention when they experience significant difficulties eating or weight loss-indicating advanced functional impairment-by which point treatment is often limited in effectiveness (13). Therefore, developing an effective tool tailored for community settings to identify older adults at high risk for oral frailty early on is crucial for enabling preventive interventions, delaying functional decline, and enhancing quality of life in later years.

Currently, the assessment of oral frailty largely relies on detailed clinical examinations conducted by specialists or comprehensive evaluation scales incorporating multiple subjective questionnaires (14) (e.g., Oral Frailty Index-8, Oral Frailty Index-6). While these methods demonstrate high validity, their dependence on professionals and time-consuming nature limit their large-scale application and promotion in routine community health management, where resources are relatively scarce and the population base is vast. Although studies have identified multiple risk factors for oral functional decline, including age, sex, number of remaining teeth, masticatory capacity, and grip strength (15, 16), no community-specific risk prediction model has yet been developed that integrates multidimensional information-such as sociodemographics, behavioral habits, simplified clinical indicators, and physical function-and undergoes rigorous methodological validation. A predictive model with robust discriminatory and calibration performance can convert readily available indicators into intuitive risk probabilities, enabling community healthcare providers to conduct rapid and efficient risk stratification. This facilitates optimized resource allocation and drives a shift from “universal management” to “precision prevention.” Given this, this study aims to systematically identify risk factors associated with oral frailty. Based on cross-sectional survey data from China’s community-dwelling older adult population, we will construct a scientific, simple, and practical community-based oral frailty risk prediction model for the older adults. Its predictive efficacy and generalization ability will be evaluated through internal validation. This study seeks to provide a quantitative tool for early screening of oral health in community-dwelling older adults, offering evidence-based support for developing targeted individual and group intervention strategies, ultimately serving the national strategy for healthy aging.

## 2 Methods

### 2.1 Research design and population

This study is a cross-sectional survey targeting community-dwelling older adults in Zigong City, Sichuan Province. Information was collected through questionnaire surveys. The entire process involved non-invasive, non-interventional procedures and posed no risk or physical harm to participating community seniors. This study was conducted in accordance with the Declaration of Helsinki and was approved by the Ethics Committee of Zigong First People’s Hospital [ethics approval number: Ethics (Research) No. 105, 2023].

This study employed convenience sampling to recruit 388 community-dwelling older adults as research subjects between October 2023 and November 2024. Inclusion criteria: (1) Community residence duration ≥6 months; (2) age ≥ 60 years; (3) no language communication barriers; (4) informed consent and voluntary participation in the study. Exclusion criteria: (1) Concurrent severe organ dysfunction (e.g., cardiac, cerebral, renal); (2) acute disease exacerbation; (3) psychiatric disorders or severe cognitive impairment; (4) malignant tumors; (5) oral diseases caused by trauma.

### 2.2 Sample size estimation

Based on the sample size estimation method (17), this study incorporated 20 variables identified through literature review. Each independent variable required 5–10 patients. A preliminary survey of 50 community-dwelling older adults revealed an oral frailty prevalence of 36%. Thus, the minimum required sample size was 334–667 cases. Accounting for a 10% attrition rate, a total of 388 community-dwelling older adults subjects were recruited. The dataset of 388 subjects was randomly split into a training set (*n* = 271) and a validation set (*n* = 117) using a random number table with a 7:3 ratio, meeting statistical requirements.

### 2.3 Variables and instruments

The general information questionnaire was designed by the research team based on literature review and expert recommendations. It includes demographic factors: sex, age, living alone, marital status, educational attainment, monthly personal income, method of medical payment, smoking habits, alcohol consumption, frequency of tooth brushing (times/day), dietary habits, number of natural teeth, and number of dentures. Disease-related factors include: number of chronic diseases and polypharmacy status.

#### 2.3.1 Oral Frailty Index-8 (OFI-8)

This scale was developed by the Japanese Dental Association (18) to assess oral function (OF) status among community-dwelling older adults. It comprises 5 dimensions and 8 items: denture use (1 item), swallowing ability (1 item), chewing ability (3 items), oral health-related behaviors (2 items), and social engagement (1 item). The total score ranges from 0 to 11, with a score ≥4 indicating OF. The scale was translated into Chinese by Chen et al. (19) and demonstrated good reliability and validity, with a Cronbach’s *α* coefficient of 0.949.

#### 2.3.2 Geriatric Self-Efficacy Scale for Oral Health (GSEOH)

This scale was adapted into Chinese by Xu et al. (20) for assessing self-efficacy related to oral health among the older adults. The scale comprises three dimensions and 20 items: oral function (9 items), oral hygiene habits (8 items), and dental visit habits (3 items). The total score ranges from 20 to 80 points, with higher scores indicating greater self-efficacy related to oral health. In this study, the Cronbach’s *α* coefficient for this scale was 0.807.

#### 2.3.3 Eating Assessment Tool-10 (EAT-10)

This scale was developed by Belafsky et al. (21) for assessing dysphagia. It comprises four dimensions (dysphagia symptoms, clinical features, psychological impact, and social impact) and 10 items. Each item uses a 5-point Likert scale ranging from “None” to “Very Severe,” scored from 0 to 4 points. The total score ranges from 0 to 40 points, with a score ≥3 indicating dysphagia. Currently, this scale is widely used to assess swallowing disorders and can effectively screen patients with swallowing difficulties (22). In this study, the Cronbach’s *α* coefficient for this scale was 0.781.

#### 2.3.4 Mini-Nutritional Assessment Short Form (MNA-SF)

This scale is primarily used to assess the nutritional status of the older adults. It was validated by Zhang et al. (23) and demonstrates good reliability and validity. The scale comprises six items (BMI index, recent weight changes, acute illness or significant psychological changes, functional capacity, neuropsychiatric disorders, and appetite), yielding a total score ranging from 0 to 14 points. A score ≥11 indicates normal nutrition, while <11 suggests malnutrition. In this study, the Cronbach’s *α* coefficient for this scale was 0.858.

#### 2.3.5 Tilburg frailty indicator (TFI)

This scale was developed by Gobbens et al. (24) primarily to assess frailty in community-dwelling older adults. It comprises three dimensions and 15 items: physical frailty (items 1–8), psychological frailty (items 9–12), and social frailty (items 13–15). Items 9, 10, 11, and 14 offer three response options: “Yes,” “Sometimes” and “No” For Item 9, selecting “Sometimes” or “No” scores 0 points, while selecting “Yes” scores 1 point. For Items 10, 11, and 14, selecting “Yes” or “Sometimes” scores 1 point, while selecting “No” scores 0 points. Items 1, 12, and 15: “No” = 1 point, “Yes” = 0 points; Items 2–8 and 13: “No” = 0 points, “Yes” = 1 point. Total score ranges from 0 to 15 points, with ≥5 points indicating frailty. Li et al. (25) demonstrated the TFI scale possesses high reliability and validity, with a Cronbach’s *α* coefficient of 0.846.

### 2.4 Data collection

Prior to the formal launch of the study, we organized a standardized training program to ensure all researchers were proficient in relevant skills. Training covered standardized procedures including GSEOH assessment, OF evaluation methods, natural tooth counting, and denture inspection. Training was conducted by senior oral physicians and clinical research staff, with assessment to validate learning outcomes. To ensure data quality, the research team strictly adhered to the protocol during data collection with clearly defined responsibilities: Dental specialty nurses conducted oral frailty assessments, natural tooth counts, and denture counts; another team member administered questionnaires. All completed questionnaires were collected immediately, underwent unified integrity verification, and were assigned unique identifiers. Prior to the survey, the purpose, significance, and confidentiality principles of the study were explained to participants. After obtaining informed consent, questionnaires were distributed on-site, with completion taking 20–35 min. If participants were unable to complete the questionnaire independently, researchers completed it on their behalf through a question-and-answer format. All collected questionnaire data were entered independently by two researchers to ensure accuracy. A total of 398 questionnaires were distributed, with 10 excluded due to data missing in over 50% of items. A total of 388 valid questionnaires were recovered, yielding a response rate of 97.5%.

### 2.5 Statistical analysis

Data analysis was performed using R software (version 4.3.2). For continuous variables meeting normal distribution, statistical description was based on mean ± standard deviation, with intergroup comparisons conducted using independent samples *t*-tests. For variables not meeting normal distribution, description was based on median [*P*25, *P*75], with intergroup comparisons performed using rank sum tests. Count data were described using counts (%). Intergroup comparisons were performed using chi-square tests, with Fisher’s exact probability test employed when chi-square tests were not applicable. Data were randomly split into training (70%) and validation (30%) sets. Potential predictors of the outcome event were screened using univariate logistic regression analysis (*p <* 0.05). For the selected variables, the Lasso (least absolute shrinkage and selection operator) logistic regression algorithm was applied to identify significant features (non-zero coefficients). Optimal parameter configuration was determined via 10-fold cross-validation. The lambda value (1 se) corresponding to the minimum distance deviation was used to determine the coefficient, and variables with non-zero coefficients were selected. The selected variables underwent further multivariate logistic regression analysis using a stepwise approach. Variables with *p <* 0.05 were incorporated into a prediction model to construct a nomogram. The nomogram underwent validation through 1,000 bootstrap samples. A calibration curve was plotted to assess model calibration, and the Hosmer–Lemeshow (H-L) test to evaluate model goodness-of-fit. Further assessment of discriminatory performance was conducted via receiver operating characteristic (ROC) curve analysis, calculating Area under the curve (AUC), sensitivity, and specificity. A decision curve analysis (DCA) was constructed to evaluate the model’s clinical utility, quantifying net benefit within the threshold probability range.

## 3 Results

### 3.1 Characteristics of the participants

**Figure 1:**
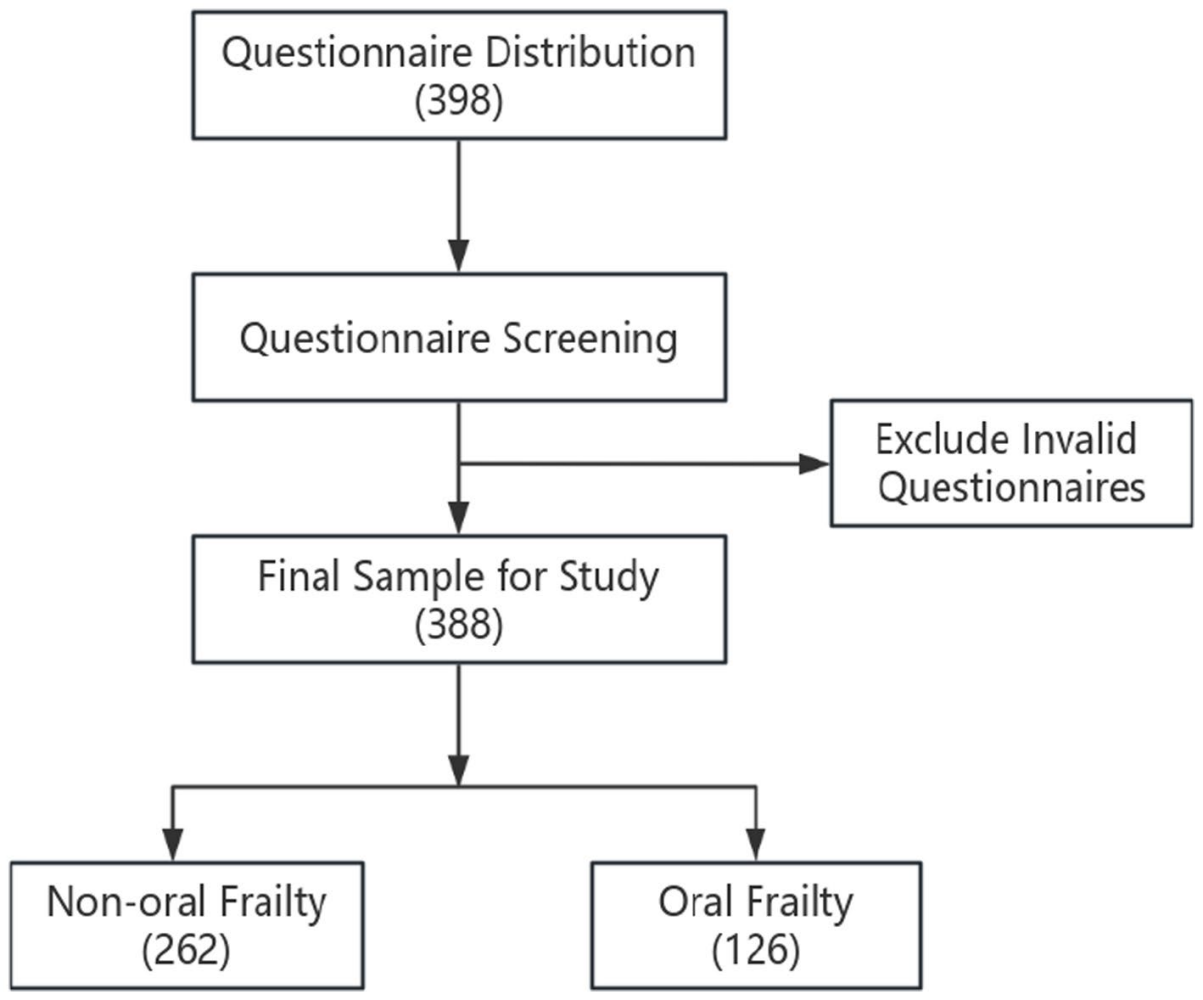
Sample screening flowchart.

This study included 388 community-dwelling older adults who met the criteria, comprising 126 cases of oral frailty and 262 cases without oral frailty (Figure 1), yielding an oral frailty prevalence rate of 32.47%. Among them, 171 were male (44.1%) and 217 were female (55.9%); 196 participants (50.5%) were aged 60–69 years, 151 (38.9%) were aged 70–79 years, and 41 (10.6%) were aged ≥80 years. No statistically significant differences were observed in general characteristics between the training and validation cohorts.

### 3.2 Univariate analysis of oral frailty

Participants were divided into non-frail and frail groups based on the occurrence of oral frailty. Statistically significant differences (*p <* 0.05) were observed between the two groups in terms of sex, age, educational attainment, marital status, living arrangements, monthly personal income, smoking status, difficulty chewing hard foods, number of chronic diseases, polypharmacy, frequency of toothbrushing, nutritional status, number of natural teeth, number of dentures, swallowing difficulties, frailty, and GSEOH scores, as shown in Table 1.

**Table 1:** Univariate analysis of oral frailty (*n* = 388).

Variables	Total (*n* = 388)	Non-oral frailty (*n* = 262)	Oral frailty (*n* = 126)	*t*/*χ*^2^value	*p*
Sex (%)				13.031	<0.001
Male	171 (44.1)	132 (50.4)	39 (31.0)		
Female	217 (55.9)	130 (49.6)	87 (69.0)		
Age (%)				20.224	<0.001
60–69	196 (50.5)	149 (56.9)	47 (37.3)		
70–79	151 (38.9)	96 (36.6)	55 (43.7)		
≥80	41 (10.6)	17 (6.5)	24 (19)		
Education (%)				20.420	<0.001
Elementary school and below	131 (33.7)	70 (26.7)	61 (48.4)		
Middle school	119 (30.7)	86 (32.8)	33 (26.2)		
Upper secondary or vocational training	93 (24.0)	75 (28.6)	18 (14.3)		
College degree or above	45 (11.6)	31 (11.8)	14 (11.1)		
Marital status (%)				6.193	0.013
Currently married	295 (76.0)	209 (79.8)	86 (68.3)		
Unmarried/divorced/widowed	93 (24.0)	53 (20.2)	40 (31.7)		
Living alone (%)				6.193	0.001
Yes	85 (21.9)	45 (17.2)	40 (31.7)		
No	303 (78.1)	217 (82.8)	86 (68.3)		
Monthly personal income (%)				19.169	<0.001
<1,000	96 (24.7)	53 (20.2)	43 (34.1)		
1,000–2,999	159 (41.0)	101 (38.5)	58 (46)		
3,000–5,000	87 (22.4)	71 (27.1)	16 (12.7)		
>5,000	46 (11.9)	37 (14.1)	9 (7.1)		
Medical payment methods (%)				3.378	0.185
New rural cooperative medical scheme	92 (23.7)	57 (21.8)	35 (27.8)		
Employee medical insurance	209 (53.9)	140 (53.4)	69 (54.8)		
Urban medical insurance	87 (22.4)	65 (24.8)	22 (17.5)		
Smoking (%)					
Yes	127 (32.7)	75 (28.6)	52 (41.3)	6.178	0.013
No	261 (67.3)	187 (71.4)	74 (58.7)		
Drinking (%)				2.900	0.089
Yes	98 (25.3)	73 (27.9)	25 (19.8)		
No	290 (74.7)	189 (72.1)	101 (80.2)		
Dietary preferences (%)				0.703	0.672
Light diet	129 (33.2)	92 (35.1)	37 (29.4)		
Salty diet	86 (22.2)	54 (20.6)	32 (25.4)		
Spicy diet	99 (25.5)	68 (26.0)	31 (24.6)		
Greasy diet	60 (15.5)	38 (14.5)	22 (17.5)		
Other diet	14 (3.6)	10 (3.8)	4 (3.2)		
Difficulty chewing hard foods				28.434	<0.001
Yes	95 (24.5)	43 (16.4)	52 (41.3)		
No	293 (75.5)	219 (83.6)	74 (58.7)		
Number of chronic diseases (%)				24.875	<0.001
0	110 (28.3)	87 (33.2)	23 (18.3)		
1–2	180 (46.4)	128 (48.9)	52 (41.3)		
≥3	98 (25.3)	47 (17.9)	51 (40.5)		
Polypharmacy (%)				15.705	<0.001
0	112 (28.9)	78 (29.8)	34 (27.0)		
1–3	176 (45.4)	132 (50.4)	44 (34.9)		
≥4	100 (25.7)	52 (19.8)	48 (38.1)		
Number of teeth brushing (%)				10.565	0.001
≤1 time/day	85 (21.9)	50 (19.1)	35 (27.8)		
≥2 time/day	295 (78.1)	212 (80.9)	91 (72.2)		
Frailty (%)				26.777	<0.001
Yes	112 (28.9)	54 (20.6)	58 (46.0)		
No	276 (71.1)	208 (79.4)	68 (54.0)		
Dysphagia (%)				21.704	<0.001
Yes	67 (17.3)	29 (11.1)	38 (30.2)		
No	321 (82.7)	233 (88.9)	88 (69.8)		
Nutritional status (%)				15.434	<0.001
Normal nutrition	327 (84.3)	234 (89.3)	93 (73.8)		
Malnutrition	61 (15.7)	28 (10.7)	33 (26.2)		
Number of natural teeth *M* (*P*25, *P*75)	20.00 (16.00, 24.00)	23.00 (20.00, 26.00)	16.00 (12, 19.00)	11.576	<0.001
Number of dentures *M* (*P*25, *P*75)	4.00 (1.00, 8.00)	3.00 (0.00, 5.00)	7.00 (5.00, 14.00)	−9.282	<0.001
GSEOH *M* (*P*25, *P*75)	50.00 (43.00, 59.00)	53.00 (46.25, 61.00)	46.00 (39.00, 51.00)	6.978	<0.001

### 3.3 LASSO regression was used to screen the predictive variables of oral frailty

**Figure 2:**
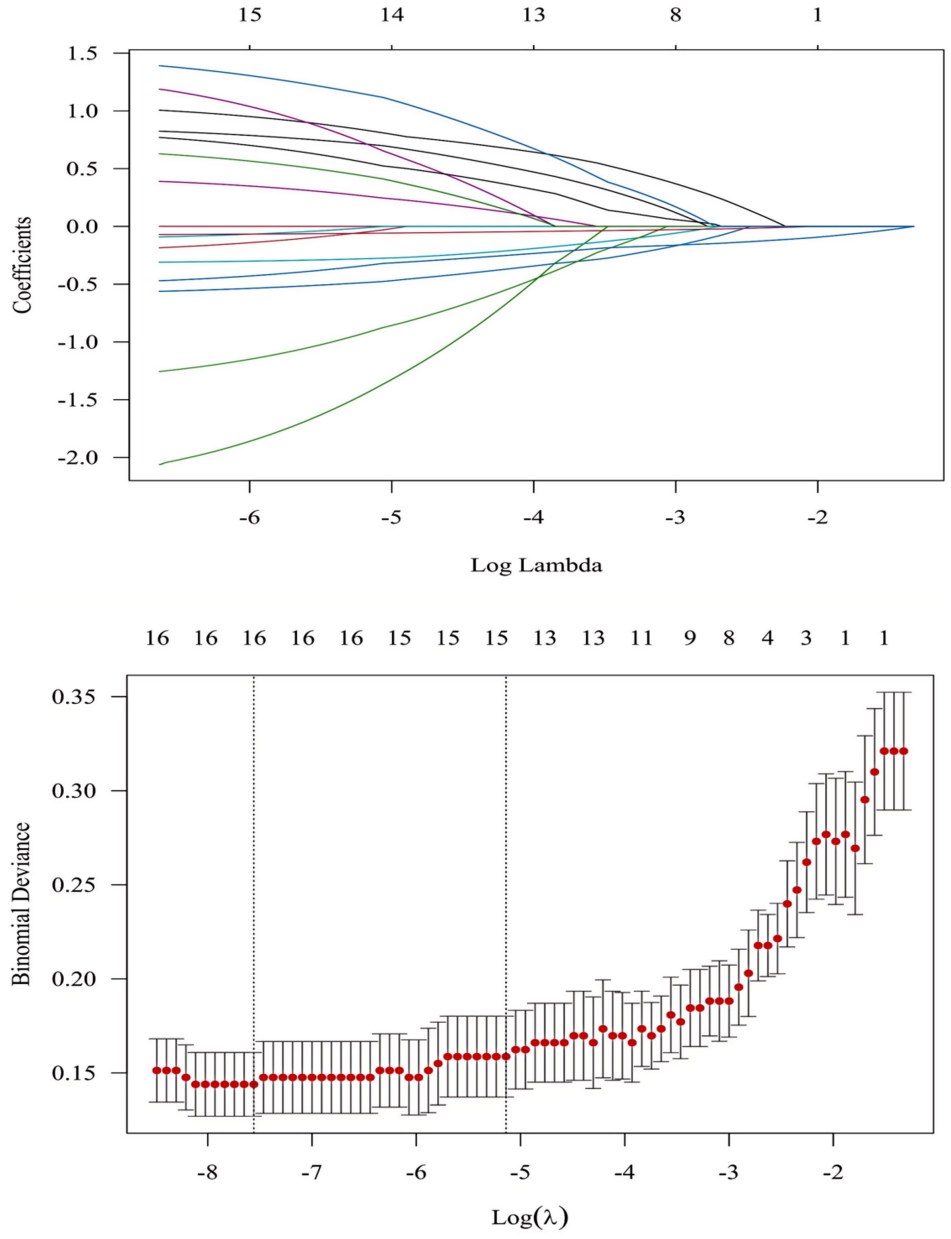
Lasso regression analysis of oral frailty.

This study employs LASSO regression to address multicollinearity issues, then utilizes stepwise regression to eliminate estimation biases from LASSO, yielding a more stable and interpretable final model. Using OF occurrence as the dependent variable and candidate variables as independent variables, Lasso regression was employed for variable selection. Lasso regression utilized 10-fold cross-validation to select the optimal *λ* parameter corresponding to the regularization parameter (*λ*) where the mean squared error (MSE) was minimized by one standard error. Results indicated an optimal *λ* value of 0.00052. At this *λ*, 15 potential predictor variables were included: sex, age, educational attainment, monthly personal income, difficulty chewing hard foods, number of chronic diseases, polypharmacy, frequency of tooth brushing, frailty, dysphagia, dietary habits, nutritional status, number of natural teeth, number of dentures, and GSEOH score (Figure 2). In this graph, the horizontal axis represents Lambda and the vertical axis represents the regression coefficient value. It can be observed that when the Lambda value decreases, the compression parameter also decreases, but the absolute value of the regression coefficient increases. Conversely, when the compression parameter increases, the absolute value of the regression coefficient decreases. The numbers at the top of the figure represent the number of independent variables in the model. As the Lambda changes, so does the number of independent variables (the graph aims to display as many results as possible corresponding to the Lambda, rather than just the result of the optimal value of the Lambda). In addition, through further cross-verification, the relationship between the mean square error and the logarithm (Lambda) was plotted, and two dashed vertical lines were drawn based on the standard error (the first dashed line represents the Lambda pair value corresponding to the minimum mean square error; the second dashed line represents the Lambda pair value corresponding to the minimum mean square error of one standard error).

### 3.4 Logistic regression analysis of oral frailty

Using OF occurrence as the dependent variable and LASSO-selected predictors as independent variables, we conducted a multivariate logistic regression analysis. Results indicated that: Sex (Male = 1, Female = 2), age (60–69 years = 1, 70–79 years = 2, ≥80 years = 3), education (elementary school and below = 1; middle school = 2; upper secondary or vocational training = 3; college degree or above = 4), number of chronic diseases (0 = 1; 1–2 = 2; ≥3 = 3), smoking (No = 0; Yes = 1), difficulty chewing hard foods (No = 0; Yes = 1), frailty (No = 0; Yes = 1), number of natural teeth, and GSEOH were independent predictors of oral frailty in community-dwelling older adults, as shown in Table 2.

**Table 2:** The logistic regression analysis of oral frailty (*n* = 388).

Variables	*β*	*S.E*	*Z*	*p*	OR (95%CI)
Sex	1.435	0.490	2.931	0.003	1.198 (1.671 ~ 11.579)
Age					
70 ~ 79	0.564	0.569	1.031	0.315	1.757 (0.586 ~ 6.272)
≥80	1.672	0.569	8.630	0.002	2.378 (1.786 ~ 15.636)
Education					
Upper secondary or vocational training	−0.281	0.577	−0.487	0.627	1.324 (0.432 ~ 4.230)
Middle school	−0.989	0.836	−1.183	0.237	0.372 (0.068 ~ 1.866)
Elementary school and below	−1.847	0.686	−2.691	0.007	0.158 (0.038 ~ 0.574)
Number of chronic diseases					
1 ~ 2	0.862	0.758	1.137	0.256	2.368 (0.562 ~ 14.241)
≥3	2.382	0.778	3.063	0.002	7.823 (2.535 ~ 26.172)
Smoking	1.620	0.464	3.495	0.001	2.289 (1.104 ~ 13.754)
Number of natural teeth	−0.489	0.077	−6.327	<0.001	0.613 (0.518 ~ 0.704)
Difficulty chewing hard foods	1.740	0.682	2.551	0.011	5.697 (1.568 ~ 27.256)
Frailty	1.575	0.596	2.643	0.008	4.833 (1.551 ~ 21.412)
GSEOH	−0.092	0.023	−4.004	<0.001	0.912 (0.869 ~ 0.952)

### 3.5 Construction and internal validation of an oral frailty risk prediction model

**Figure 3:**
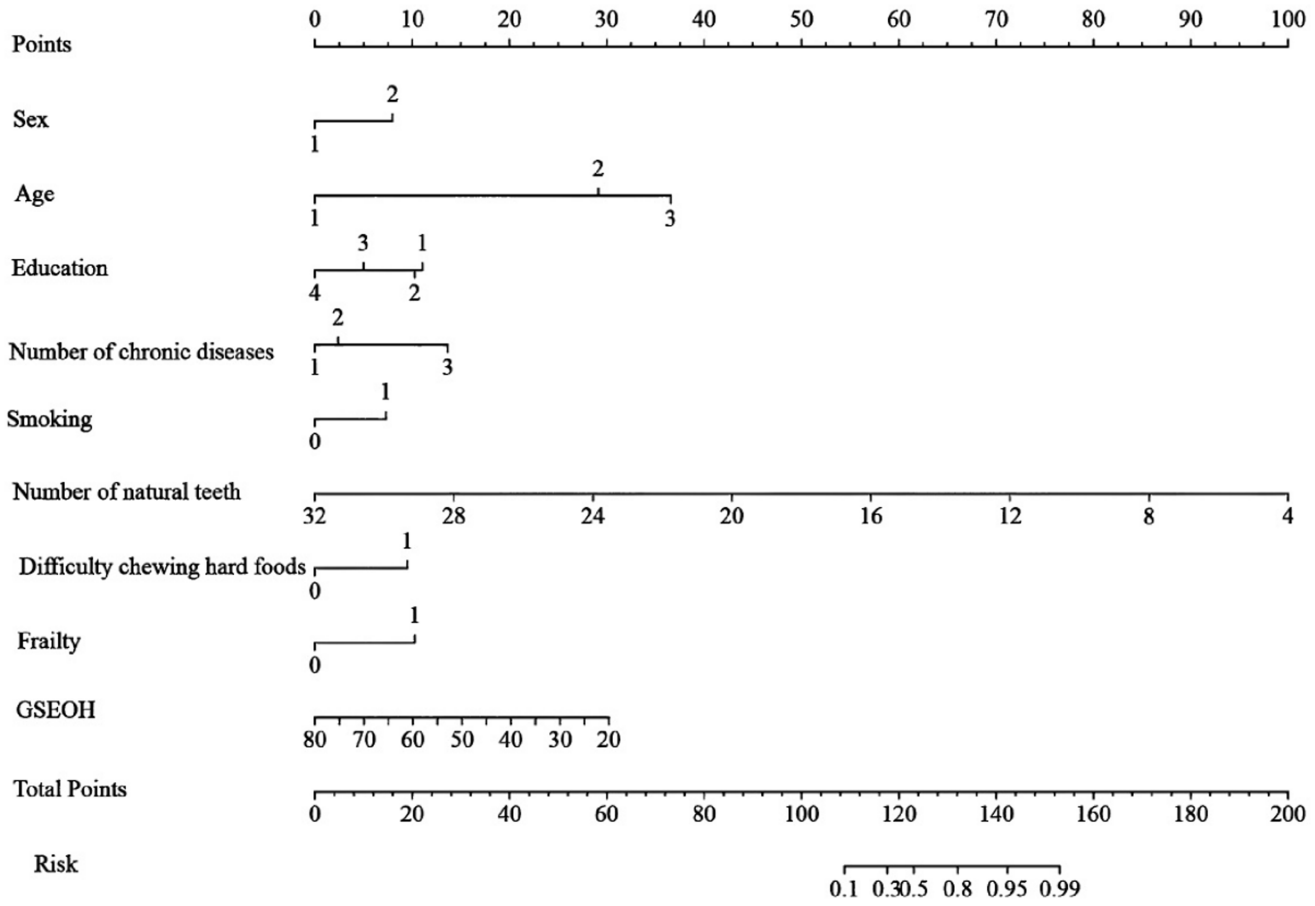
Nomogram for predicting the risk of oral frailty.

**Figure 4:**
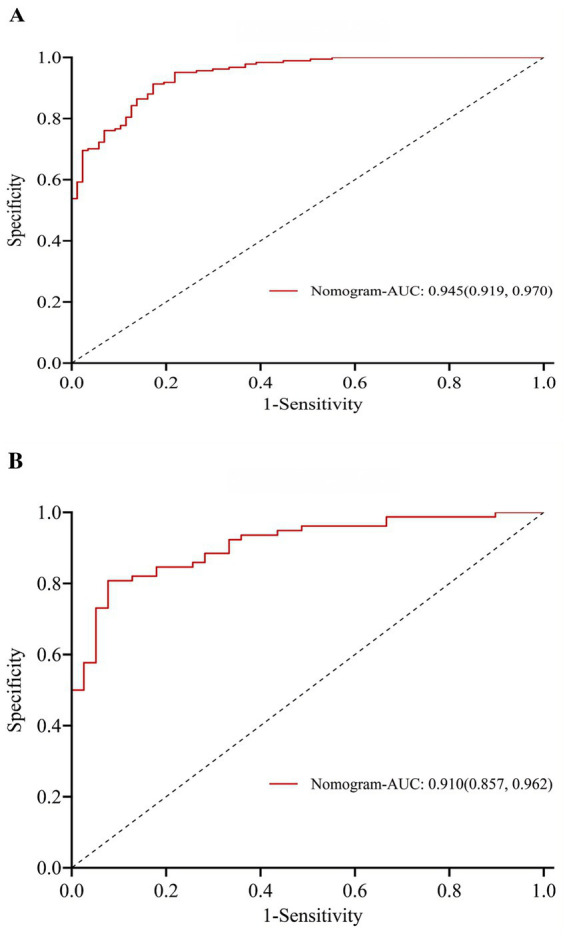
**(A)** ROC curve of the training set. **(B)** ROC curve of validation set.

**Figure 5:**
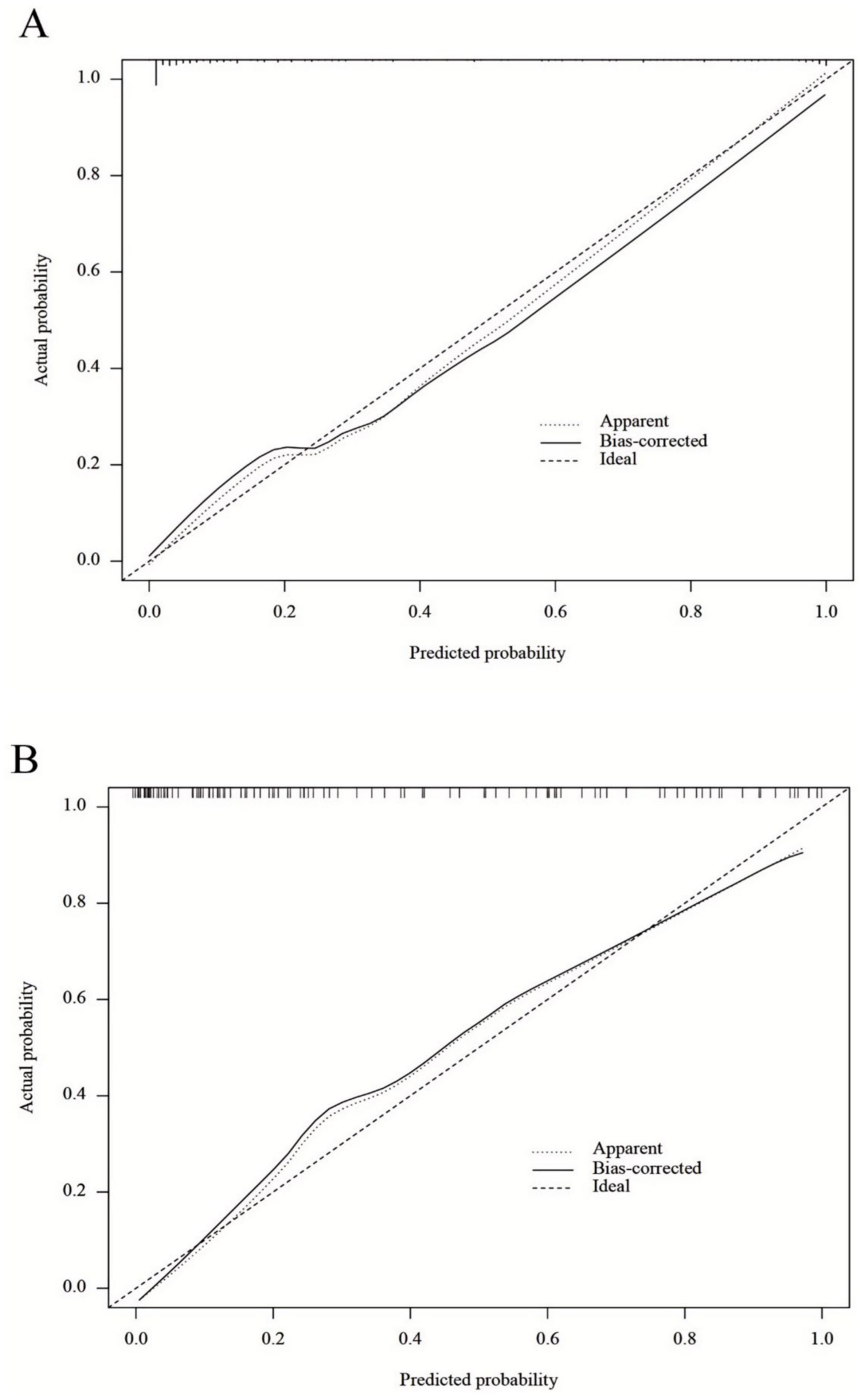
**(A)** Calibration plot for the training dataset. **(B)** Calibration plot for the validation dataset.

This study employed LASSO regression analysis to screen variables, followed by multifactorial logistic regression analysis to identify characteristic variables: sex, age, educational attainment, number of chronic diseases, smoking status, number of natural teeth, difficulty chewing hard foods, frailty, and self-efficacy related to oral health in older adults. A nomogram prediction model for OF in community-dwelling older adults was constructed (Figure 3). The H-L test results showed *χ*^2^ = 5.378, *p* = 0.915 > 0.05, indicating good model fit. The optimal cutoff value was determined to be 0.422. The training set model yielded an area under the AUC of 0.945 (95% CI: 0.919–0.970), sensitivity of 0.828, and specificity of 0.913. Internal validation revealed that the validation set model had an AUC of 0.910 (95% CI: 0.857–0.962), sensitivity of 0.923, and specificity of 0.808 (Figure 4). Calibration curves demonstrated that this regression model consistently predicted the risk of OF in community-dwelling older adults across both training and validation sets, aligning with actual risk profiles (Figure 5).

**Figure 6:**
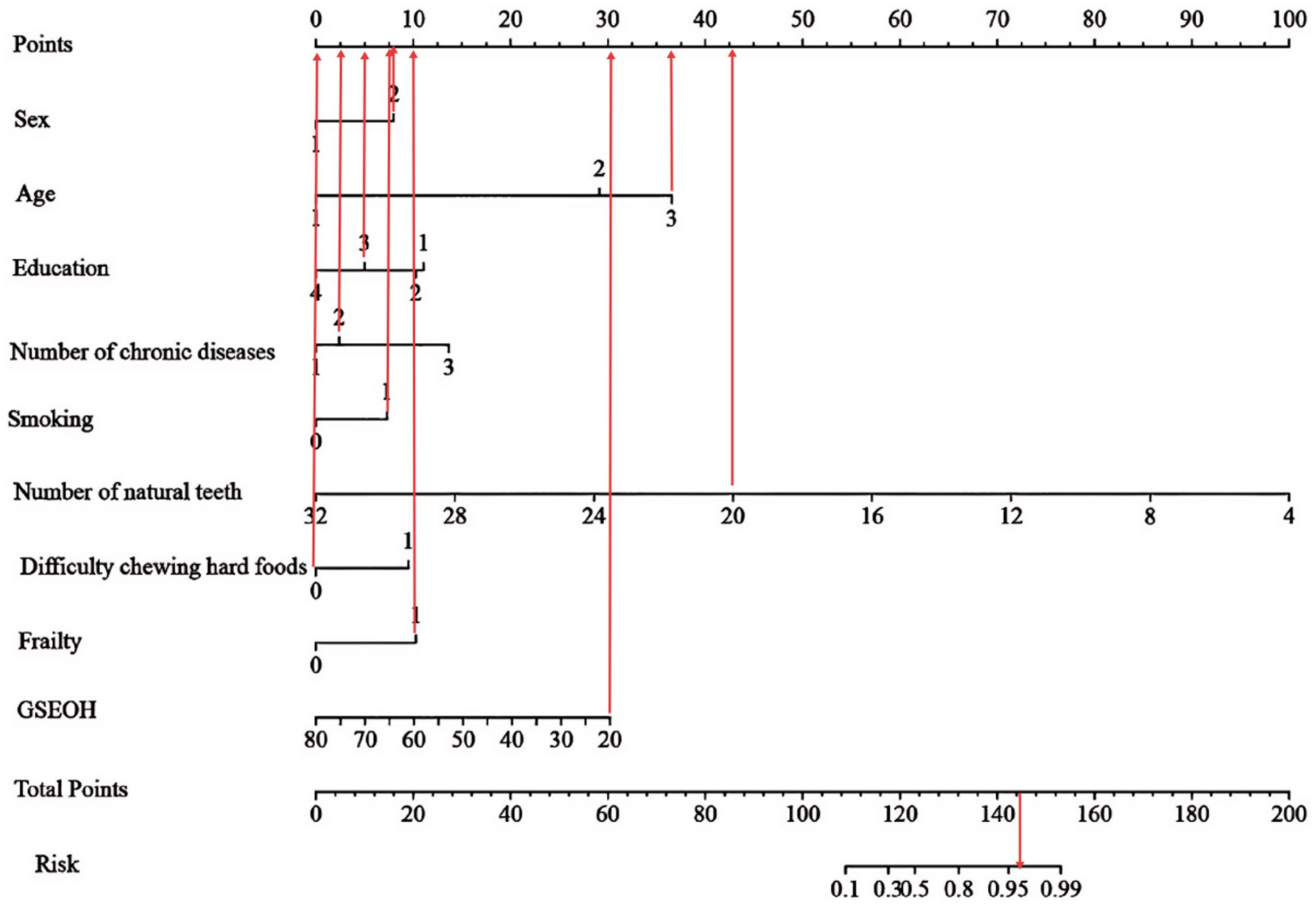
Example of clinical application of nomogram for the risk of oral frailty.

The nomogram was read according to the value of each risk factor, and the corresponding score at the top of the nomogram was obtained by the vertical line, and then the scores of all risk factors were added to obtain the total score. The corresponding risk probability can be obtained from the line of total score value at the bottom of the nomogram. Figure 6 shows an example of the clinical application of Nomogram in this study: consider an 80-year-old female community resident with a high school education, 1 ~ 2 chronic conditions, a smoking history, 20 natural teeth, no difficulty chewing hard foods, frailty, and a GSEOH score of 20. Her corresponding scores on the “Points” axis are 8, 36.25, 5, 5, 7.5, 42.5, 0, 10, 30, totaling 144.25 points. A vertical line drawn at 144.25 points on the “Total Points” scale corresponds to the “Risk” axis, indicating an approximate 96% risk of OF.

### 3.6 Evaluation of the clinical efficacy of a risk prediction model for oral frailty

**Figure 7:**
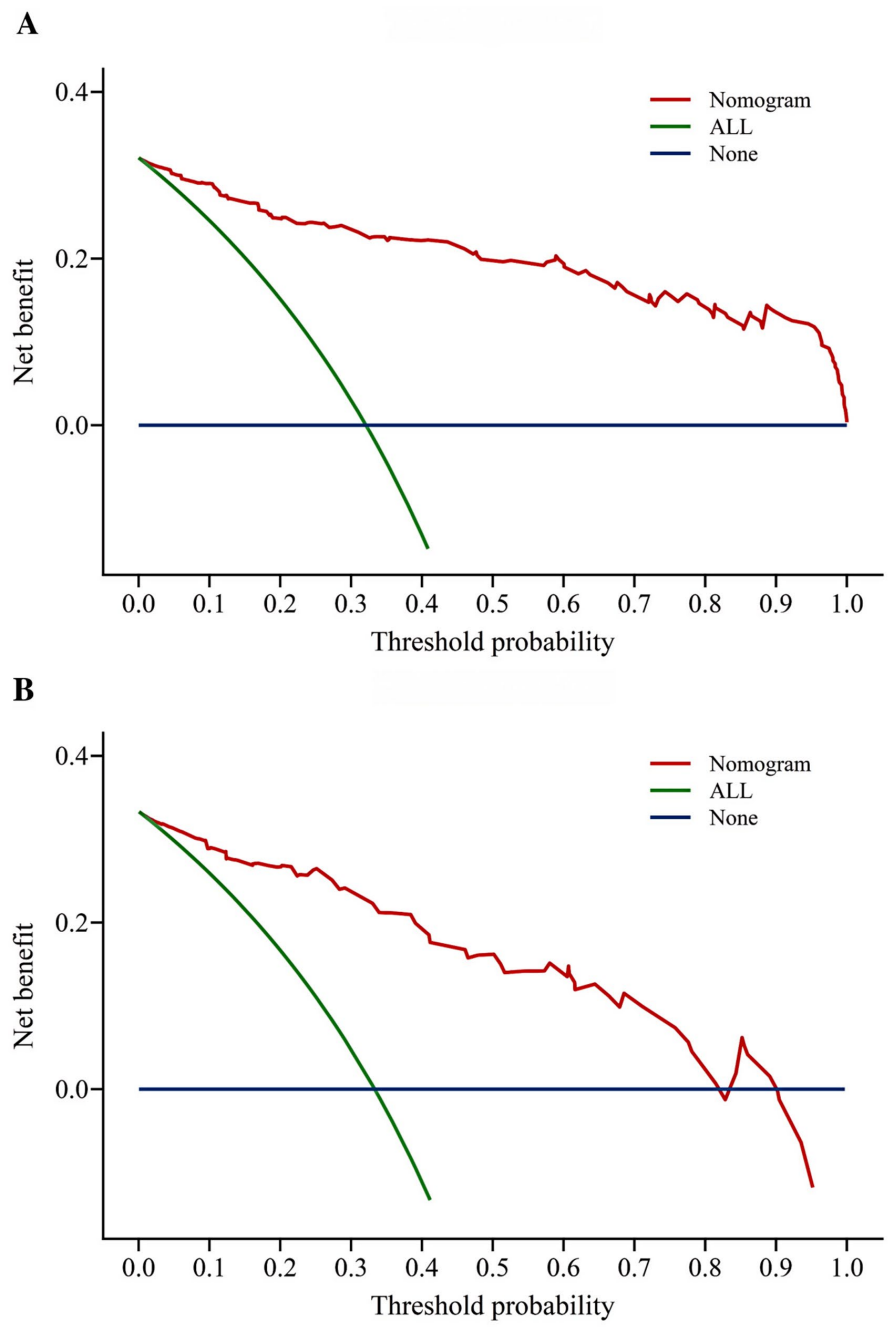
**(A)** DCA curves for the training dataset. **(B)** DCA curves for the validation dataset.

In this study, the decision curve analysis method was used to evaluate the benefit degree of the community older adults, and DCA decision curves of the training set and the validation set were drawn. According to the DCA decision analysis curve results OF the training set and the validation set, the risk prediction model of OF the community older adults constructed in this study has good clinical practical value (Figure 7). The ordinate represents the net benefit rate after subtracting the harms from the benefits. The green slash and blue horizontal lines represent the two extreme cases. In this study, the green slash indicates that all community older adults experienced OF and all received interventions, the net benefit is the backslash with a negative slope, and the blue horizontal line indicates that all patients did not experience OF and did not receive any interventions, and the net benefit is zero. The red line represents the net benefit of the prediction model based on this study. The DCA decision analysis curves of the training set and the validation set showed that the red line representing the model was not close to the two extreme case lines, and the overall trend was toward the upper right corner, indicating that the model constructed in this study had good clinical application value and certain clinical benefits.

## 4 Discussion

The results of this study indicate that the prevalence of OF among community-dwelling older adults is 32.47%. This finding is consistent with the study by Hangjia et al. (11), but higher than the 28% reported by Zhou et al. (26) and the 24% reported by Li et al. (27). Concurrently, the present findings are lower than the 34% reported by Huang et al. (28) in the general older adult population and the 45.2% reported by Chen et al. (29) among older adult hemodialysis patients. Similarly, the 45.9% reported by Yang et al. (30) among older adult patients with type 2 diabetes and the 47.8% reported by Ma et al. (31) among older adult stroke patients were both higher than the findings in this study. Furthermore, Li et al. (32) reported an OF incidence as high as 57.58% among patients undergoing cancer chemotherapy. This disparity in prevalence rates may be attributed to differences in assessment tools across studies and variations in chronic disease characteristics. For instance, in diabetic patients, a hyperglycemic environment not only promotes the proliferation of pathogenic bacteria in the oral cavity but also impairs leukocyte function. This reduces the patient’s ability to resist infection, making them more susceptible to severe periodontitis and adversely affecting oral health (30). For hemodialysis patients, the treatment process, fluid intake restrictions, and medication side effects can reduce salivary flow rate. This facilitates microbial accumulation and adhesion in the oral cavity, weakening saliva’s protective role for teeth and mucosa and increasing the risk of oral vulnerability (29). Stroke patients experience impaired oral function due to neurological deficits, muscle weakness, and dysphagia, further increasing susceptibility (31). Among chemotherapy patients, drug-induced reduced salivary secretion severely diminishes the oral cavity’s self-cleaning capacity, leading to plaque accumulation and creating a vicious cycle: “reduced saliva → plaque buildup → worsening caries (especially root caries).” This dental damage ultimately results in decreased chewing efficiency and malocclusion, directly accelerating the progression of oral frailty (32–35). Studies have shown that compared with the older adults without OF, the older adults with OF have 2.4, 2.2, 2.3, 1.1 and 2.2 times higher risks of physical frailty, sarcopenia, disability, disability and even death (4, 18). Shirobe et al. (36) conducted a 12-week oral function management program for community-dwelling older adults. The intervention included oral motor exercises, mouth opening training, tongue pressure resistance training, rhythmic training, and chewing training. Results demonstrated a significant reduction in OF symptoms, indicating that comprehensive oral rehabilitation exercises can effectively treat OF. Therefore, early identification of OF risk in community-dwelling older adults and timely intervention can help reduce or delay the onset and progression of OF, extend life expectancy, improve quality of life, and promote healthy aging.

The results OF this study show that older adult women in the community have a higher risk of OF than men, which is similar to the results of previous studies (11). This may be attributed to the fact that permanent teeth develop earlier in girls than in boys during childhood, resulting in prolonged periods of chewing wear and bacterial erosion over time (37). Additionally, the gums serve as a target organ for estrogen. After menopause, declining estrogen levels and bone calcium loss lead to alveolar bone resorption, reduced saliva secretion in the oral mucosa, and increased vascular permeability. These changes contribute to conditions such as dry mouth, dental caries, and periodontal disease (38), making women more susceptible to oral functional disorders. The risk of OF increases with age among community-dwelling older adults. This may be attributed to several age-related changes: decreased alkaline phosphatase activity in periodontal ligament cells, gradual reduction in salivary secretion, weakened periodontal ligament cell regeneration and osteogenic activity, demineralization and softening of cementum, physiological gingival recession, and dental nerve degeneration (39). These factors collectively diminish oral resistance in the older adults, and diminished self-cleaning capacity, thereby increasing the risk of OF. This study also found that community seniors with lower educational attainment face a higher risk of OF. This may stem from poorer oral health management practices and attitudes among less educated patients, who may rarely seek proactive oral care or knowledge about disease prevention. Inadequate understanding of brushing techniques, flossing, toothbrush replacement schedules, periodontal disease, and other risk factors affecting oral health contributes to declining oral health status and weakened oral muscle strength. Additionally, smoking increases the risk of OF, consistent with findings by Zhong et al. (40). Long-term smoking facilitates tartar accumulation on periodontal surfaces, reducing the resistance of periodontal tissues (41). Furthermore, nicotine in tobacco slows oral blood circulation and diminishes the oral cavity’s self-healing capacity (40). Therefore, healthcare professionals or community workers should help community seniors recognize the harms of smoking and encourage or assist them in quitting.

This study found that community-dwelling older adults with ≥3 chronic diseases had a higher risk of developing OF compared to those without chronic diseases. This may be attributed to the fact that older adults with multiple chronic conditions require long-term use of various medications, many of which can cause oral dryness (4). Oral dryness, in turn, can lead to oral mucosal inflammation, oral ulcers, dental caries, and increased bacterial proliferation in the oral cavity, thereby compromising oral health and elevating the risk of OF. The number of natural teeth is also a risk factor for OF in community-dwelling older adults. Dental count serves as a crucial indicator of oral health in the older adults. It is well-established that tooth loss, particularly posterior tooth loss and edentulism, diminishes masticatory force and impacts the masticatory muscles. Maintaining healthy natural teeth, preventing oral diseases, and avoiding tooth loss can protect individuals from the effects of OF (42). Therefore, it is recommended to maintain natural teeth through regular cleaning, use dentures when teeth are lost, and persist with oral chewing function training. Community-dwelling older adults experiencing difficulty chewing hard foods face a higher risk of oral frailty (43). This may stem from individuals with chewing difficulties avoiding foods like meat, fruits, and vegetables due to eating challenges (11). Over time, this leads to inadequate nutrient intake, weakened oral chewing function, oral muscle atrophy, and swallowing disorders-all factors that elevate the risk of oral frailty. Traditional dental treatments have often focused solely on prosthetic restoration without addressing declining oral chewing function (44). For older adults to live longer, maintaining oral function, oral health, and sustained chewing and eating abilities is essential (45, 46). Therefore, to achieve this goal and better promote OF prevention, it requires not only the involvement of oral health professionals but also multidisciplinary collaboration among other healthcare specialists. This collaboration should develop feasible and effective intervention measures to reduce or delay the onset and progression of OF.

The results of this study show that among older adult people living in the community, there is a strong association between frailty and an increased risk of OF. Research indicates that physical frailty is typically associated with muscle weakness and reduced physical activity (47). When frailty occurs in older adults, it may lead to decreased mobility or restricted social interaction and contact with others, thereby reducing opportunities for verbal communication. This can affect the body, resulting in decreased activity of the pharyngeal muscles, perioral muscle groups, and tongue, which may ultimately be related to impaired oral function (7, 48). Additional research indicates that oral frailty itself may impair an individual’s ability to eat, leading to inadequate nutritional intake and thereby exacerbating physical frailty (49). A bidirectional relationship may exist between physical frailty and oral frailty; however, this study adopted a cross-sectional design, and it is still impossible to confirm the causal relationship between the two. Therefore, healthcare providers are advised to consider combining exercise interventions with nutritional management when guiding frail older adults. This approach aims to improve physical frailty while positively influencing the reduction of OF risk. This study found that among community-dwelling older adults, low oral health self-efficacy was significantly associated with a higher risk of oral frailty. Oral health self-efficacy may positively influence oral health by enhancing health awareness and improving oral health behaviors. However, it is noteworthy that the deterioration of oral health status in the older adults—such as tooth loss and reduced masticatory function leading to oral frailty—may itself undermine their confidence in maintaining oral health, thereby diminishing self-efficacy. Given that the cross-sectional design of this study precludes establishing causality, prospective research could be designed in the future to further explore this bidirectional relationship. At the practical level, healthcare providers or community workers can utilize oral health self-efficacy as a convenient indicator for identifying oral frailty risk, prioritizing older adults with low self-efficacy scores. Through comprehensive care, health promotion, and science-based education, they can help enhance participants’ oral health knowledge, attitudes, and self-efficacy, thereby improving oral health behaviors and ultimately supporting enhanced quality of life in older adults.

This study developed a predictive model for OF risk in community-dwelling older adults and created a nomogram. The design and validation phases fully considered the practical realities of community healthcare, aiming to provide a convenient and efficient risk screening tool for older adults in community settings. From the perspectives of target users, application scenarios, and implementation costs, this model demonstrates strong potential for clinical translation and practical value for widespread adoption. Healthcare professionals at community health service centers serve as the primary users of this tool. They can utilize the model during routine chronic disease follow-ups or geriatric health examinations to rapidly conduct preliminary oral health screening and risk stratification. For social workers or medical staff conducting home-based older adult care assessments, this tool serves as an effective supplement for quantitative risk evaluation. Furthermore, the model’s predictive factors—such as age, difficulty chewing hard foods, and number of chronic conditions—are non-invasive indicators obtainable through simple questioning. A single assessment is estimated to take only 5–10 min, imposing no additional burden on daily workflows. Furthermore, none of the predictors require complex dental equipment or specialized expertise. After approximately 20 min of brief training, community health workers can proficiently master the scoring criteria and risk interpretation methods. This provides scalable and operational practical tools for the early identification and stratified intervention of oral frailty among the older adults in the community.

## 5 Conclusion

The oral frailty risk prediction model developed in this study incorporates nine predictive factors: age, sex, education, number of natural teeth, difficulty chewing hard foods, number of chronic diseases, smoking, frailty, and self-efficacy related to oral health in older adults. The model demonstrates good discrimination and calibration, effectively identifying high-risk individuals for oral frailty among community-dwelling older adults. It provides a concise, practical tool for early screening of oral frailty risk in community settings. By comprehensively considering factors across physiological function, behavioral, and psychosocial dimensions, the model suggests that prevention and intervention for oral frailty require integrated, multidisciplinary, and multidimensional strategies. Future research may explore targeted interventions based on these risk predictors, thereby providing evidence-based support for early management and promotion of oral health among the older adult population, ultimately enhancing their overall quality of life.

## 6 Limitations

The community-based geriatric oral frailty risk prediction model developed in this study demonstrates excellent screening efficacy. The visualized nomogram created provides an intuitive and convenient tool, quantifying complex predictive indicators into a straightforward individual risk score, thereby offering a practical instrument for early identification of high-risk older adults in communities. However, this study also has certain limitations. First, the cross-sectional study design can only reveal associations between factors, not establish causality or clarify the temporal sequence of predictive variables. Future longitudinal studies are needed for further validation. Second, the sample originates solely from communities in Zigong City, Sichuan Province, potentially introducing selection bias that limits the model’s generalizability and representativeness. Third, some variables (e.g., chewing difficulties, smoking) relied on self-reporting, potentially introducing recall bias. Fourth, while the number of chronic diseases was included as an important predictor, it was not specified by disease type (e.g., diabetes, stroke, cardiovascular disease), which may affect the model’s comprehensiveness. Finally, although our model demonstrated excellent performance in internal validation with a high AUC value, models constructed from single-center retrospective data often exhibit overoptimism. The model’s high performance may depend on the specific patient population distribution at this center. Furthermore, this study lacks independent external validation. Although we employed rigorous Bootstrap resampling to assess model stability, these methods cannot fully substitute for external validation using data from diverse populations and centers, potentially leading to inflated AUC values. Therefore, we recommend that future research obtain external validation data through multicenter data collection and prospective studies to rigorously evaluate the model’s generalization ability and further confirm its reliability and clinical utility.

## Data Availability

The raw data supporting the conclusions of this article will be made available by the authors, without undue reservation.
